# Accurate proteome-wide protein quantification from high-resolution ^15^N mass spectra

**DOI:** 10.1186/gb-2011-12-12-r122

**Published:** 2011-12-19

**Authors:** Zia Khan, Sasan Amini, Joshua S Bloom, Cristian Ruse, Amy A Caudy, Leonid Kruglyak, Mona Singh, David H Perlman, Saeed Tavazoie

**Affiliations:** 1 Department of Computer Science, Washington Rd, Princeton University, Princeton, NJ 08544, USA; 2 Lewis-Sigler Institute for Integrative Genomics Washington Rd, Princeton University, Princeton, NJ 08544, USA; 3 Current address: Department of Human Genetics, South Ellis Avenue, University of Chicago, Chicago, IL 60637, USA; 4 Department of Molecular Biology, Washington Rd, Princeton University, Princeton, NJ 08544, USA; 5 Current address: Illumina Inc., Towne Centre Drive, San Diego, CA 92121, USA; 6 Cold Spring Harbor Laboratory, Bungtown Rd, Cold Spring Harbor, NY 11724, USA; 7 Current address: Donnelly Centre for Cellular and Biomolecular Research, University of Toronto, King's College Circle, Toronto, Ontario, M5S 1A1, Canada; 8 Department of Ecology and Evolutionary Biology, Washington Rd, Princeton University, Princeton, NJ 08544, USA; 9 Howard Hughes Medical Institute, Washington Rd, Princeton University, Princeton, NJ 08544, USA; 10 Princeton Mass Spectrometry Center, Washington Rd, Princeton University, Princeton, NJ 08544, USA; 11 Current address: Department of Biochemistry and Molecular Biophysics, 116th Street and Broadway, Columbia University, New York, NY 10027, USA

## Abstract

In quantitative mass spectrometry-based proteomics, the metabolic incorporation of a single source of ^15^N-labeled nitrogen has many advantages over using stable isotope-labeled amino acids. However, the lack of a robust computational framework for analyzing the resulting spectra has impeded wide use of this approach. We have addressed this challenge by introducing a new computational methodology for analyzing ^15^N spectra in which quantification is integrated with identification. Application of this method to an *Escherichia coli *growth transition reveals significant improvement in quantification accuracy over previous methods.

## Background

Experimental methods for proteome-wide quantification using liquid chromatography coupled mass spectrometry (LC-MS) rest heavily on computational methods that analyze mass spectra for peptide and protein quantification [1]. Computational analysis typically relies on two main components: (1) a peptide identification algorithm that assigns amino acid sequences to fragmentation spectra by searching a database of theoretical peptides obtained by an *in silico *digest of an organism's proteome; and (2) a quantification algorithm that collects peaks across mass spectra in the form of extracted ion chromatograms (XICs) over the duration of chromatographic elution of peptides.

These algorithms have been shown to produce robust and accurate peptide quantification results across a wide range of quantification strategies. They are an important part of the data analysis employed in a widely used experimental approach called stable isotope labeling by amino acids in cell culture (SILAC) [2,3]. In most typical applications of this approach, a sample in which stable isotope-labeled amino acids are incorporated metabolically by an organism is compared to an unlabeled sample from a different experimental condition. Because both samples are combined prior to protein extraction, the samples are subjected to the same extraction, sample handling, digestion, chromatography, and ionization conditions. This eliminates much of the technical variation between individual samples. Consequently, the final relative abundance measurements, in the form of ratios of the areas of paired XICs from both the unlabeled and labeled peptides, are highly accurate. To ensure that only labeled amino acids are incorporated into proteins, amino acid auxotrophs are typically employed in conjunction with labeled amino acids, rendering this strategy unavailable for prototrophic microorganisms, such as wild-type bacteria and yeast.

^15^N-labeling through the metabolic incorporation of a single source of labeled nitrogen provides an alternative strategy for labeling virtually all nitrogen in expressed proteins of prototrophs and auxotrophs [4]. ^15^N-labeling has three notable advantages over labeling with specific amino acids. First, all peptides are labeled, irrespective of their amino acid composition, and therefore any endogenous peptide or peptides produced by any chosen endoprotease can be used for quantification. The use of multiple endoproteases has been shown to significantly increase the number of distinct, non-overlapping peptides identified per protein [5]. Second, the label is not subject to the complications of metabolic conversion of amino acids [6]. Such label conversion is a particular problem in yeast, where arginine is efficiently converted into proline [7]. Third, labeled ammonium salts are much less expensive than labeled amino acids and are efficiently used as a nitrogen source by many microorganisms, including *Escherichia coli *and both budding and fission yeasts.

Despite these advantages, labeled amino acids have been used in favor of ^15^N-labeling for proteome-wide quantification in microbes [8]. Amino acid labels typically produce a small number of distinct mass differences between pairs of XICs, allowing all XICs and XIC pairs to be found prior to database search. In contrast, ^15^N-labels produce mass differences that depend on the length and composition of a peptide, complicating the detection of such pairs. Existing methods for ^15^N quantification rely on peptide database search of fragmentation spectra to locate paired peaks in parent mass spectra [6,9-12]. Statistically significant matches to a merged database of both unlabeled and ^15^N-labeled peptides provide the labeling status of a fragmentation spectrum and an amino acid sequence from which nitrogen composition can be determined. During quantification, these methods find peaks within parent mass spectra that fall within an interval centered at the precursor retention time and precursor mass-to-charge ratio (*m/z*) of the peptide-assigned fragmentation spectrum. Then, a mass difference, computed using the nitrogen composition and precursor charge, is used to find peaks in parent mass spectra that correspond to the XIC with the opposite labeling status. By examining only peaks in parent mass spectra near a fragmentation spectrum and at the expected mass difference, existing methods use only a small fraction of the information in all the parent mass spectra to distinguish signal from noise, resulting in noisy peptide quantifications. Therefore, the lack of a robust quantification method significantly limits the wide use of ^15^N-labeling for proteome-wide quantification in prototrophic microbes.

We have developed a novel approach for ^15^N quantification that finds all XICs as a first step, reliably using all the available information in all the parent mass spectra. Once XICs are found, our method pairs these XICs by using two databases of labeled and unlabeled intact peptide fragment masses and peptide nitrogen counts. Then, pair membership allows the method to determine the label status of a fragmentation spectrum and search the appropriate peptide database. Additionally, the XIC pair provides a constraint on the nitrogen composition, allowing the search to limit the peptides scored against any given fragmentation spectrum. In an experiment to compare the proteome of stationary phase *E. coli *with that of exponentially growing cells, we show that our method significantly improves the quantitative accuracy of protein abundance ratios obtained from ^15^N mass spectra.

## Results and discussion

**Figure 1:**
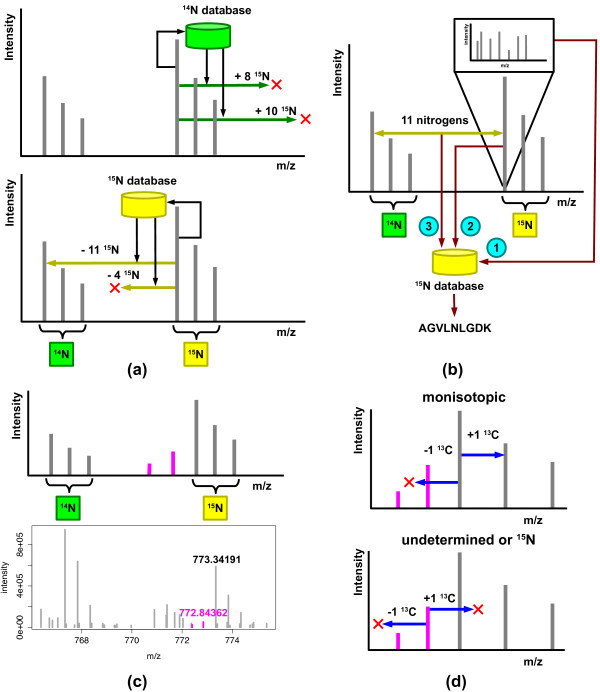
**Approach for peptide quantification using ^15^N mass spectra**. **(a) **The method uses accurate mass and charge measurements to search a ^14^N-unlabeled database and a ^15^N database of intact peptide masses and nitrogen counts obtained by *in silico *digestion of an organism's proteome. The number of nitrogens in each of the returned peptides is used to examine a limited number of mass differences designated by arrows. On finding an unambiguous ^14^N-^15^N pair, the method labels each member of the pair as originating from either the unlabeled or the ^15^N-labeled sample. **(b) **During peptide identification, a fragmentation spectrum (1) associated with a member of the pair is searched against an unlabeled database or ^15^N-labeled database of peptides based on the assigned label status. Note that only one member of the peptide pair needs to have an associated fragmentation spectrum. The monoisotopic masses of the peptides (2) and their nitrogen composition (3) are used to limit the search space of peptides scored against the spectrum. The intensity of each member of a ^14^N-^15^N pair is used to derive a peptide ratio. **(c) **Top, a population of incompletely labeled peptides can generate a complex isotope distribution pattern for ^15^N-labeled peptides. The peaks in purple, in order of decreasing *m/z*, correspond to peptides lacking one and two labeled nitrogens. Bottom, even in highly enriched samples, such peaks can be found in real mass spectra as illustrated for a +2 charge peptide, LGFFETVDTDTQR from *E. coli *protein yaeT (b0177). **(d) **For a range of charge states, monoisotopic XICs can be paired with an XIC with an additional ^13^C, but lack any XICs detected at a negative ^13^C shift (top). In contrast, ^15^N XICs will lack both XICs at negative and positive ^13^C shifts (bottom).

Our method finds XICs prior to database search of fragmentation spectra using techniques we have previously developed [13]. From the accurate mass and charge information assigned to a monoisotopic XIC, our method searches entries in unlabeled and ^15^N peptide databases and uses the returned nitrogen counts to examine a limited number of ^15^N mass differences (Figure 1a). After unambiguously pairing XICs, the method designates each XIC as originating from either the unlabeled or the ^15^N-labeled sample. This process is repeated for all monoisotopic XICs until no more XICs can be paired.

During peptide identification, the method iterates through paired XICs that have an associated fragmentation spectrum. Based on the label assigned to an XIC, our method searches the fragmentation spectrum against an unlabeled database or a database of ^15^N-labeled peptides. In addition to the mass of the peptide, this search uses the discovered constraint on the nitrogen composition of a peptide from the pairing phase to limit the search space of peptides scored for identification (Figure 1b). Last, the peptide expression ratios are obtained from the areas of paired XICs. Remaining, unpaired monoisotopic XICs that have associated fragmentation spectra are assigned a labeling status based on the highest scoring search result from the unlabeled or the labeled database.

A critical element of our approach is the identification of monoisotopic XICs. This process is complicated by the presence of incompletely labeled peptides. Even in highly enriched ^15^N-labeled samples, a population of incompletely labeled peptides can generate a set of XICs that have a more complex isotope distribution pattern than the unlabeled sample (Figure 1c). To distinguish the monoisotopic XIC from this incompletely labeled population, we leverage the precision of modern high-resolution mass spectrometers. Our approach rests on the observation that the difference *d *= 0.00631994439 Da that exists between the 0.99703489341 Da shift between ^15^N isotopes and the 1.00335483781 Da shift between ^13^C isotopes for charge +1 peptides allows these incompletely labeled XICs to be distinguished from ^13^C XICs. If the precision *p *of a high-resolution instrument approaches 1 part per million (ppm), this shift can be distinguished at up to (*d */ *p*) × 106 = 6,319 in *m/z*, above the maximum measured in a typical bottom-up proteomics experiment. At a charge state of +4, this shift can be distinguished at up to 1,579 in *m/z*. Examples of such shifts can be found in real spectra, as illustrated for a pair of peaks generated by unlabeled and labeled populations of a +2 charge peptide, LGFFETVDTDTQR from *E. coli *protein yaeT (b0177; Figure 1c, bottom). Here, the error between the theoretical shift and the observed shift between the peaks generated by the population of peptides lacking a single ^15^N and the fully labeled population is 4.4 ppm and 0.3 ppm for ^13^C and ^15^N, respectively. An instrument with precision of 4 ppm would be sufficient to identify this shift as originating from a ^15^N isotope, as opposed to a ^13^C isotope. To leverage the precision of modern high-resolution instruments, our method labels XICs as monoisotopic if, for a range of charge states, no XIC is found at a negative ^13^C shift and an XIC is found at a positive ^13^C shift (Figure 1d, top). An XIC is labeled as undetermined or ^15^N if no XICs are found at both negative and positive ^13^C shifts for a range of charge states (Figure 1d, bottom). Additionally, if a ^15^N or undetermined XIC was selected for fragmentation, its associated fragmentation spectrum can also be transferred to the nearest monoisotopic XIC at a positive ^15^N shift to increase identification rates [14]. Also, in experimental samples where high enrichment levels cannot be obtained, due to limitations in labeling efficiency and impurities in the nitrogen source, the intensity information in ^13^C and ^15^N XICs found by our high-precision approach can be leveraged to generate ratios that are adjusted for significant populations of these partially labeled peptides [14].

In order to evaluate our approach, we performed an experiment to quantitatively compare the proteomes of stationary and exponential phase *E. coli *(see Materials and methods). We selected these two conditions because the transition to stationary phase is well-characterized, and the expression levels of a large number of proteins during this transition are known to differ over a wide range [15]. Stationary and exponential phase cells were harvested from ^15^N-labeled and unlabeled media, respectively. Cell lysate was prepared, combined, and rendered into peptides by trypsin digestion, and then subjected to two-dimensional nano-flow LC-MS analysis.

**Figure 2:**
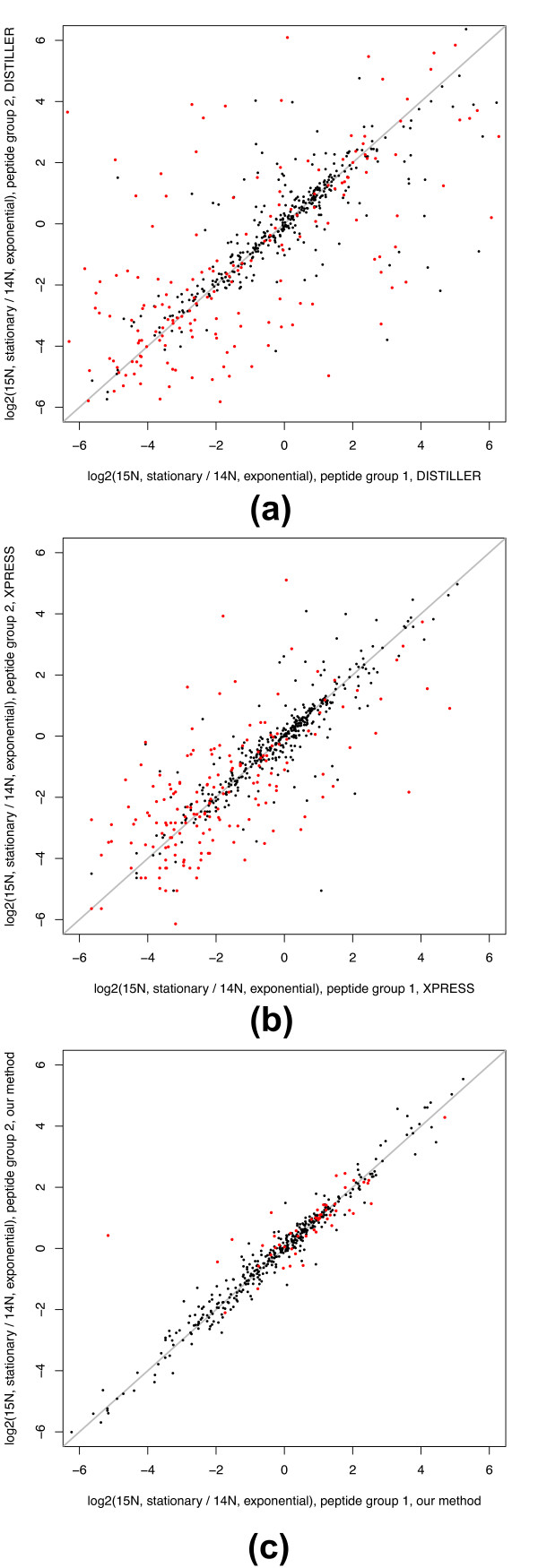
**Comparison with XPRESS and Mascot Distiller**. **(a-c) **Correlation of log_2 _ratios between distinct peptides from the same protein obtained by Mascot Distiller (a) [16], XPRESS (b) [10], and our method (c). Peptides from each protein were randomly assigned to two groups for this comparison of log_2 _ratios of peptides in group 1 versus log_2 _ratios of peptides in group 2. Multiple measurements (for example, charge states) of the same peptide, when present, were collated and given a median log_2 _ratio prior to forming the peptide groups. Black points designate proteins identified and quantified by both methods, and red points designate proteins identified and quantified that were specific to each method. The diagonal gray line designates perfect correlation.

We analyzed the resulting MS data, comparing our approach to the method employed by XPRESS and Mascot Distiller v.2.3.2. Both methods rely on finding XIC pairs by searching fragmentation spectra against a merged database of both unlabeled and ^15^N-labeled peptides [10,16]. The parameters for XPRESS and Distiller were selected to be appropriate for the precision and mass accuracy of the data. In order to evaluate the methods, we examined the consistency among ratios measured from distinct peptides belonging to the same protein. We expected that distinct peptides from the same protein would be determined to have approximately the same ratios of stationary phase to exponential measurements. For proteins with two or more distinct quantified peptides, we randomly assigned the peptides from each protein into one of two groups and computed the median log_2 _ratio measured for peptides in the two groups. The two ratios, from peptide group 1 and peptide group 2, were correlated for XPRESS and Distiller as well as our method (Figure 2). The significantly higher Spearman's correlation for our method (0.96 our method, 0.84 for XPRESS, and 0.67 for Distiller) indicated that our method produced significantly better quantitative measurements and peptide identifications.

Both Distiller and XPRESS reported more proteins (*N *= 664 Distiller; *N *= 554 XPRESS) with two or more quantified peptides than our method (*N *= 424). This difference in total number of quantified proteins reported by the methods could either be due to differences in sensitivity of accurately determined proteins, or differences in accuracy of quantification. To investigate this, we observed that the Spearman's correlation for proteins (*N *= 385 Distiller; *N *= 373 XPRESS) quantified by two or more peptides by our method and each of Distiller and XPRESS independently was higher for our approach (0.98 our method; 0.79 Distiller; 0.89 XPRESS). Next, we examined the correlation for proteins specific to XPRESS (*N *= 181), Distiller (*N *= 226), and to our method (*N *= 51). For these proteins, we computed significantly higher correlations for our method (0.87 our method; 0.49 Distiller; 0.65 XPRESS), suggesting that erroneous pairing of unrelated peaks and subsequent mis-quantification of the assigned peptides contribute to the inferior performance of XPRESS and Distiller.

We additionally examined the raw mass spectra corresponding to the XICs of the one outlier protein yihX (b3385) reported by our method (Figure 2c). The outlier was a peptide with the sequence VLGAWSDLTR. From parent mass spectra, it appeared that an unknown peptide with nearly the same mass co-eluted with both the labeled and unlabeled versions of VLGAWSDLTR, leading to incorrect detection of XICs. However, the exact cause of the problem was difficult to discern by eye since the peaks belonging to the XICs were of very low intensity.

In order to evaluate the reproducibility of our method, we conducted a label-swap experiment. We independently cultured two identical sets of stationary phase and exponential phase cells differing only by which culture was grown in the ^15^N labeled media. Analysis of both biological replicates by our method enabled quantification of 539 proteins and 1,985 peptides at a stringent 1% peptide-level false discovery rate (FDR). High correlations across protein and peptide ratios (Spearman's 0.96 and 0.94, respectively) from the two biological label-swap replicates indicated that the analysis was highly reproducible (Figure S1a,b in Additional file 1).

The label swap experiment allowed us to investigate the apparent increase in variance from XPRESS for negative log_2 _peptide ratios (Figure 2b). When we conducted the same analysis using XPRESS, we found the reverse pattern of increasing variance towards positive log_2 _ratios (Figure S2a in Additional file 1). No such increasing variance was observed from our method (Figure S2b in Additional file 1). These results indicate the increasing variance in the negative range in XPRESS is linked to the growth condition of the cells and may reflect that XPRESS is sensitive to the differing expression levels of proteins in the two experimental conditions. Additionally, our method reported slightly more (*N *= 408) proteins with two or more quantified peptides than XPRESS (*N *= 399) for the label-swap. Correlations across peptide ratios were higher for our method overall (0.97 our method; 0.84 XPRESS), for proteins in common (0.97 our method; 0.85 XPRESS), and proteins specific to each method (0.94 our method; 0.79 XPRESS). This additional result provides further evidence that indicates our method provides more accurate quantitative measurements and peptide identifications than XPRESS.

The label-swap experiment also allowed us to examine the number of multiply coupled monoisotopic XICs and unpaired monoisotopic XICs present in ^15^N mass spectra: 221 monoisotopic XICs in the forward experiment and 254 monoisotopic XICs in the label-swap experiment were multiply coupled. For comparison, 5,556 monoisotopic XIC pairs were found in the forward experiment and 5,224 monoisotopic XIC pairs were found in the label-swap experiment. Unpaired monoisotopic XICs could be generated by several experimental factors: a missed pairing, a multiply coupled XIC that was left unpaired, a protein that is expressed in only one of the conditions, an expression difference outside the detectable range, or contaminant compounds. As all of these factors contribute unpaired XICs, we found in the forward experiment 28,426 monoisotopic XICs of which 17,093 were left unpaired and in the label-swap experiment 31,256 monoisotopic XICs of which 20,554 were left unpaired.

We additionally used the identified XIC pairs from the label-swap experiment to estimate the precision of our data set. Using the nitrogen composition of the peptide sequence assigned to a pair, we calculated an expected theoretical shift between the two XICs. By computing the difference between the measured shift between the XICs and the expected theoretical shift, we created a histogram of precision measurements in parts per million. The mean precision was 0.151 ppm and the standard deviation was 0.987 (Figure S3 in Additional file 1). Our estimate of precision indicates that the difference between ^15^N isotopes and ^13^C isotopes can be detected for charge states +1 and +2 for about 90% of the XICs for the entire *m/z *range measured. For peptides with charge states +3 and +4, this *m/z *limit reaches approximately 1,301 and 976, respectively.

In order to deepen our quantitative analysis of the proteomes to include proteins of lower abundance, we subjected our original protein sample to additional fractionation steps. First, the sample was partitioned based on protein solubility into two fractions: high solubility and low solubility. Next, peptides derived from each of these two protein-level fractions were further fractionated using strong cation exchange (SCX) chromatography (see Materials and methods). These additional fractionation steps enabled quantification of 2,320 distinct proteins, 14,605 distinct peptides, and 29,658 quantified XIC pairs at a 1% peptide-level FDR (Additional file 2 and Materials and methods).

To evaluate our method on this deeper survey of the proteome, we examined the correlations between the ratios measured for different peptides from the same protein (Figure S4a in Additional file 1). High correlations (Spearman's 0.96) indicate that our method could accurately identify and quantify distinct peptides from the same protein to measure the fold-change of the whole protein. In addition, we correlated ratios between charge +2 and charge greater than +2 isoforms of the same peptide (Figure S4b in Additional file 1). Although the differing charge states produce very different fragmentation spectra, the peptide ratios from the two charge states were highly correlated (Spearman's 0.98), confirming the high degree of accuracy in both our quantifications and our assignments for each peptide. Next, we examined the intra-experiment variability of the ratios reported by the method. From the ratios of distinct peptides from each protein, we calculated a protein level median coefficient of variation (CV) of 18.4% (Figure S5a in Additional file 1). We also calculated a peptide level median CV of 11.9% from ratios of peptides observed in multiple fractions or with differing charge states (Figure S5b in Additional file 1). These low CVs at both the protein level and peptide level indicate high quantification accuracy. In summary, the depth and accuracy of this analysis illustrates the utility of our method for large-scale proteomics studies.

We further used the data from the additional fractions to evaluate the sensitivity and specificity gained by using the discovered constraint on the nitrogen composition of a peptide from the XIC pairing phase to limit the search space of peptides scored for tandem mass spectrometry (MS/MS) identification. We independently analyzed each of the 24 fractions in two ways: with our method's nitrogen composition constraint imposed and with it deactivated (Additional file 3). When our method was applied to the data, the MS/MS score cutoff required to maintain a constant 1% FDR was significantly less for all charge states, indicating statistically significant gain in sensitivity (paired *t*-test, *P *< 3.69 × 10^-10 ^for charge states +1 and +2 and *P *< 1.35 × 10^-11 ^for charge states +3 or greater). When our method was deactivated, the highest scoring search results above the 1% FDR threshold were free to have nitrogen compositions that did not match the composition discovered by XIC pairing. As these are likely incorrect peptide identifications, they can be removed by simple filtering. After filtering, the total number of search results and quantifications returned by our method on the same data was significantly higher (Wilcoxon signed rank, *P *< 9.68 × 10^-6^). This additional result indicates that our method provides a statistically significant gain in specificity.

**Figure 3:**
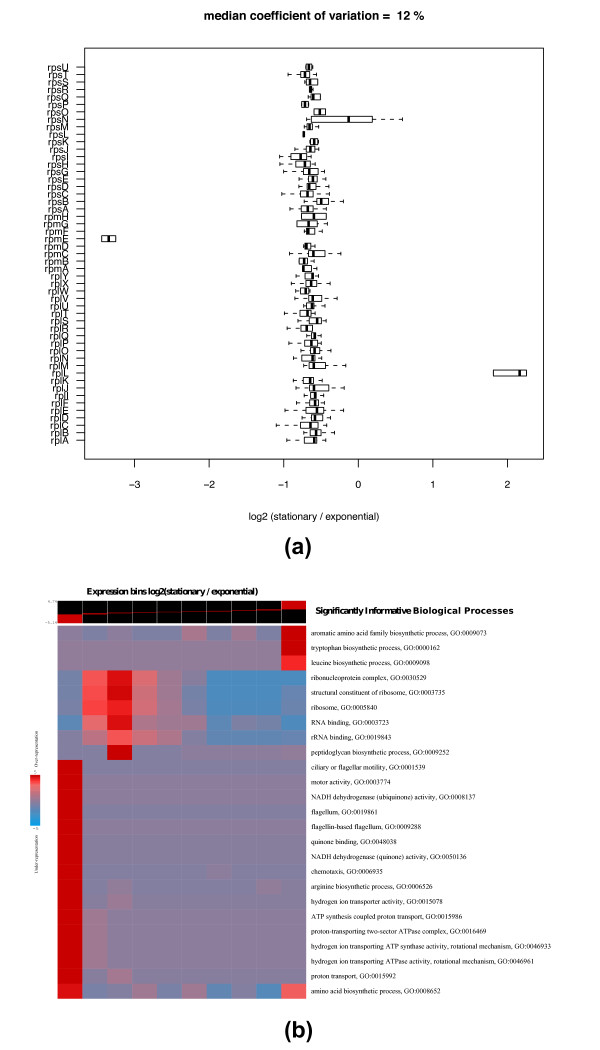
**Ribosomal protein co-regulation and functional enrichment analysis of protein expression ratios**. **(a) **Box and whisker plot of the log_2 _ratios of stationary over exponential phase for 51 ribosomal proteins from the low solubility protein fraction. Boxes designate the quartiles. The thick centerline designates the median. Thin lines illustrate the range. **(b) **A graphical representation of the functional enrichment analysis method from [19] applied to protein expression ratios comparing stationary to exponential phase of growth in *E. coli *computed by our method. In this representation, rows correspond to significantly informative pathways and columns correspond to 10 equally populated expression bins of log_2 _ratios (stationary over exponential). Colors indicate pathway over or under-representation levels across the expression bins. Red designates (in log_10_) over-representation of a particular pathway in any expression bin, whereas, blue designates under-representation.

Next, we investigated whether the quantitative output of our method captured expression level changes of proteins known to differ between exponential and stationary phase. Globally decreased rates of translation and ribosome biosynthesis characterize stationary phase growth [17]. In strong agreement with expectation, we observed that 49 of 51 quantified ribosomal proteins were down-regulated by a similar amount upon transition into stationary phase (Figure 3a). Another known feature of stationary phase is that cells adapt to higher cell density and nutrient limitation by inducing the expression of many stress proteins, including starvation-induced DNA protection protein, Dps, and osmotically inducible proteins, OsmC and OsmY [15]. This is orchestrated by several regulators, including the RNA polymerase sigma factor, RpoS [18]. We observed elevated expression levels of each of these proteins in stationary phase cells (Additional file 2), in agreement with previous results.

Finally, to examine whether our measured protein expression differences on a global level accurately characterize the differences between exponential phase and stationary phase cells, we conducted a functional enrichment analysis that bins expression differences based on their magnitudes and directions and determines which biological processes are over- or under-represented in these bins (Figure 3b) [19]. Our results demonstrated that, in addition to cessation of ribosome biosynthesis [17], we captured many other adaptive physiological changes across major biological processes that are known features of stationary phase in *E. coli*. These include repression of flagellum biosynthesis and motility associated processes [20] and down-regulation of proteins involved in oxidative metabolism [21].

## Conclusions

We have introduced a new computational analysis method that significantly improves the accuracy of proteome-wide protein quantification from ^15^N mass spectra. The method combines two ideas, one to use pairing information to constrain database search, earlier proposed in [2], and another to pair XICs using nitrogen counts, earlier proposed in [22], to obtain an integrated approach for identification and quantification. Using protein samples from a well-characterized growth transition in *E. coli*, we show that the new method enables the collection of high-quality quantitative data for large-scale proteomics experiments, which is validated by numerous metrics, including its consistency with previously published observations. More broadly, the method allows researchers in the fields such as microbiology to leverage the robustness and accuracy of metabolic labeling without the experimental limitations and complications of amino acid labeling approaches. Because of this practical advantage, we anticipate that our method will provide a valuable tool for proteome-wide protein quantification.

## Materials and methods

### Strain and media

*E. coli *K-12 MG1655 was used for all bacterial experiments in this work. Bacterial cells were grown in M9 minimal media with 0.4% w/v glucose as carbon source. The M9 media contained 12.8 g Na_2_HPO_4_-7H_2_O, 3 g KH_2_PO_4_, 0.5 g NaCl, 11 mg CaCl_2_, 241 mg MgSO_4_, and 1 g NH_4_Cl (with either ^14^N or ^15^N) per liter. ^15^N-ammonium chloride was purchased from Cambridge Isotope Laboratories (Andover, MA, USA). Growth conditions: overnight cultures of *E. coli *MG1655 were diluted into fresh M9 media (1:500) and shaken at 250 rpm at 37°C. Cultures were harvested at OD600 values of 0.2 and 0.9 for exponential and early stationary phase samples, respectively.

### Protein extraction

We harvested 100 ml of exponentially growing cells (in ^14^N media) and 22 ml of stationary phase cells (in ^15^N media) by centrifugation and these were frozen in an ethanol-dry ice bath. The pellets were re-suspended in 1 ml of B-PER bacterial protein extraction buffer (Pierce, Rockford, IL, USA), pooled together, and vortexed vigorously for 1 min. The mixture was centrifuged at 13,000 rpm for 5 min. The supernatant (high solubility fraction) was collected and frozen in an ethanol-dry ice bath. The pellet was re-suspended in 2 ml of 1:10 diluted B-PER reagent (PIERCE). The suspension was centrifuged and washed one more time with 1:10 diluted B-PER reagent. The pellet was re-suspended in 1 ml of Inclusion Body Solubilization Reagent. The suspension was vortexed for 1 min, shaken for 30 min, and sonicated to further break the cellular structures. Cell debris was removed from the suspension by centrifugation. The supernatant was frozen in an ethanol-dry ice bath (low solubility fraction).

### Mass spectrometry sample preparation

The low solubility and high solubility protein fractions were subjected to buffer exchange, thiol reduction and alkylation, and tryptic digestion by the filter-aided sample preparation (FASP) procedure [23]. Peptides were desalted using a home-packed capillary reversed-phase column (500 μm internal diameter × 20 cm, POROS 10R2 C18 resin) using a Harvard syringe pump, and eluted directly onto a home-packed capillary strong cation exchange column (500 μm internal diameter × 45 cm, POROS SCX resin ), which was connected to the outlet of the reversed-phase column to minimize sample loss. SCX fractionation of peptides was conducted using a Dionex Ultimate NanoLC capillary HPLC system (Dionex, Sunnyvale, CA, USA), using a gradient from a 75%:25% mix of buffers A:B to 100% buffer B (buffer A, 7 mM KH_2_PO4, pH 2.65, 30% acetonitrile (ACN); buffer B, 7 mM KH_2_PO_4_, 350 mM KCl, pH 2.65, 30% ACN) over an 80-min period at flow rate of 10 μl/min, followed by column stripping and reconditioning for 10 min in buffer C (50 mM K_2_HPO_4_, 500 mM NaCl, pH 7.5) and water. SCX fractions were collected every 5 min and were pooled into 12 fractions of roughly equivalent peptide abundance according to the integration of their UV absorbance (λ = 214 nm) values during the course of separation.

### Mass spectrometry data acquisition

Fractions were desalted using StageTip micro-scale reversed-phase chromatography [24], then subjected to reversed-phase nano-LC-MS and MS/MS performed on a nano-flow capillary high pressure HPLC system (Eksigent, Dublin, CA, USA) coupled to an LTQ-Orbitrap hybrid mass spectrometer (ThermoFisher Scientific, San Jose, CA, USA). Sample concentration and desalting were performed online using a trapping capillary column (200 μm × approximately 30 mm, packed with 5 μm, 100 Å Magic AQ C18 material; Michrom, Auburn, CA, USA) at a flow rate of 7 μl/min for 3.5 min, while separation was achieved using an analytical capillary column (75 μm × approximately 20 cm, packed with 3 μm, 100 Å Magic AQ C18 material; Michrom) terminating in a pulled sprayer tip, under a linear gradient of A and B buffers (buffer A, 3% ACN/0.1% formic acid; buffer B, 97% ACN/0.1% formic acid) over 180 min at a flow rate of approximately 0.5 μl/min. Electrospray ionization was carried out at 2.5 kV, with the LTQ heated capillary set to 200°C. Full-scan mass spectra were acquired in the Orbitrap in the positive-ion mode over the *m/z *range of 300 to 1,800 at a resolution of 60,000. MS/MS spectra were simultaneously acquired using the LTQ for the seven most abundant multiply charged species in the full-scan spectrum having signal intensities of > 1,000 NL (Thermo). Dynamic exclusion was set such that MS/MS was acquired only once for each species over a period of 120 s.

### Peptide identification and quantification

We used a database of peptide sequences for *E. coli *K-12 from the UniprotKB, which was built by *in silico *tryptic digest to obtain two databases of labeled and unlabeled peptide masses and nitrogen counts. XICs and their associated fragmentation spectra were detected using methods we previously developed [13]. Monoisotopic XICs were paired in order of decreasing intensity. During pairing, the mass determined from the mass-to-charge ratio (*m/z*) and charge z obtained from ^13^C isotope spacing was used to search both databases. A positive or negative shift of 0.99703489341 × N/z was examined for candidate XICs for each returned nitrogen count N for the unlabeled and labeled database, respectively. The *m/z *of the candidate XIC was allowed to vary between a ± 3 ppm window. Also, the pairing allowed the position of a candidate XIC to vary between the start and end time of the current XIC to compensate for differences in co-elution time known to occur due to ^15^N-labeling. If only a single candidate XIC was found, the current XIC and the candidate XIC were paired, and a labeling status and nitrogen count were assigned to any associated fragmentation spectra. Based on labeling status, fragmentation spectra were searched against an unlabeled or labeled tryptic peptide database using the fragmentation model from the Open Mass Spectrometry Search Algorithm [25] and scored according to (M/L) Σ log_2 _(I_m_), where L is the number of peaks in the theoretical spectrum, M is the number of matched experimentally observed peaks, and I_m _is the intensity of the m-th peak that matched a theoretical spectrum peak. This modified score was used to leverage intensity information in spectra and assure near Gaussian distribution of the scores. Prior to scoring, peptides with matching precursor masses but differing nitrogen counts were filtered. FDR was estimated using a q-value approach and concatenated reverse decoy database [26,27]. XICs that have fragmentation spectra without a corresponding XIC pair, or an ambiguous pair, can be assigned an identification and labeling status based on database search of a labeled and ^15^N-labeled database.

We used the implementation of XPRESS in Trans Proteomic Pipeline (v.4.4.1) [12]. The mass tolerance for XPRESS was set to 0.05 Da. All tryptic peptides were searched using a precursor mass window of ± 10 ppm at a stringent FDR of 1%. A single missed cleavage was allowed during peptide database construction.

### Availability

We have implemented our method in an open source software system for LC-MS data analysis that provides an extremely fast and highly efficient implementation of this method [13,28]. A tutorial and all the raw mass spectra analyzed in this manuscript are available for download at [29] and at Proteome Commons [30] with the following Tranche Hash: 4b3VyisnIV6ZXYHRRebqqLxSpgy45DrDVqJojES07+m8JrGaL8RshZx6y/XFZNYSU1iL3VJY7mwhi3XvxhESYXOpBaYAAAAAAAACuQ = =

## Abbreviations

ACN: acetonitrile; CV: coefficient of variation; FDR: false discovery rate; LC-MS: liquid chromatography coupled mass spectrometry; MS/MS: tandem mass spectrometry; ppm: parts per million; XIC: extracted ion chromatogram; SCX: strong cation exchange; SILAC: stable isotope labeling by amino acids in cell culture.

## Authors' contributions

SA conducted the growth transition experiment and the Gene Ontology enrichment analysis, conceived the study in its design and coordination and helped to draft the manuscript. JB conducted the initial pilot experiments from which the method was designed, conceived the study in its design and coordination and helped to draft the manuscript. ZK conducted the LC-MS data analysis, designed and implemented the method with input from MS, conceived the study in its design and coordination and helped to draft the manuscript. CR contributed the idea of comparing to XPRESS. DP conducted the mass spectrometry experiments and data acquisition, conceived the study in its design and coordination and helped to draft the manuscript. AC, LK, MS and ST conceived the study in its design and coordination and helped to draft the manuscript. All authors read and approved the final manuscript for publication.

## Supplementary Material

Additional file 1 **Supplementary figures**. Additional figures referred to in the text. Click here for file

Additional file 2 **Peptide ratios for fractionated data set**. Raw peptide ratios reported by our method for the solubility and SCX fractionated *E. coli *protein samples. Click here for file

Additional file 3 **Sensitivity and specificity results**. False discovery rate (FDR) 1% score thresholds and total number of quantified peptides with and without our method for limiting peptides scored during MS/MS database search based on nitrogen composition applied to a data set. Click here for file
