# Supplementary material for: Accurate proteome-wide protein quantification from high-resolution 15N mass spectra
Source: Genome Biol. 2011 Dec 19;12(12):R122. doi: 10.1186/gb-2011-12-12-r122 (PMC3334617; doi:10.1186/gb-2011-12-12-r122)
Supplement: Additional file 1 — Supplementary figures. Additional figures referred to in the text. [file gb-2011-12-12-r122-S1.PDF]

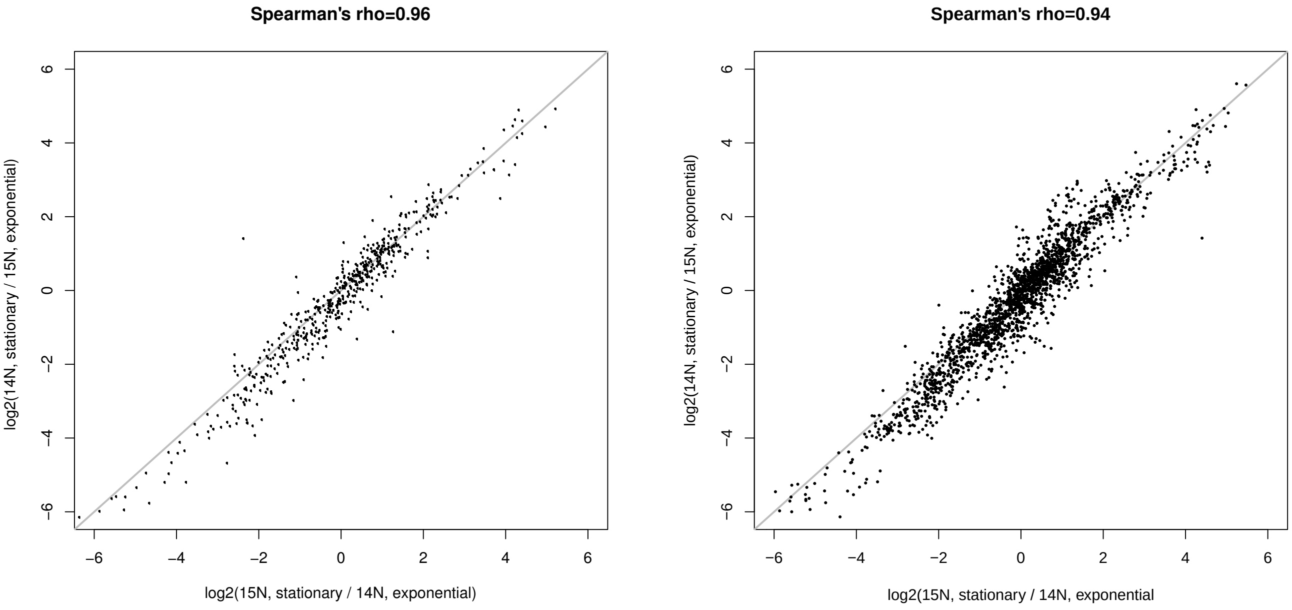

(a) (b)

Figure S1

Correlation of $\log_2$ (a) protein and (b) peptide ratios computed from an unfractionated, label-swap experiment in which two biological replicates with heavy or light labeling were produced to compare *E. coli* cells grown in stationary phase to those grown in exponential phase.

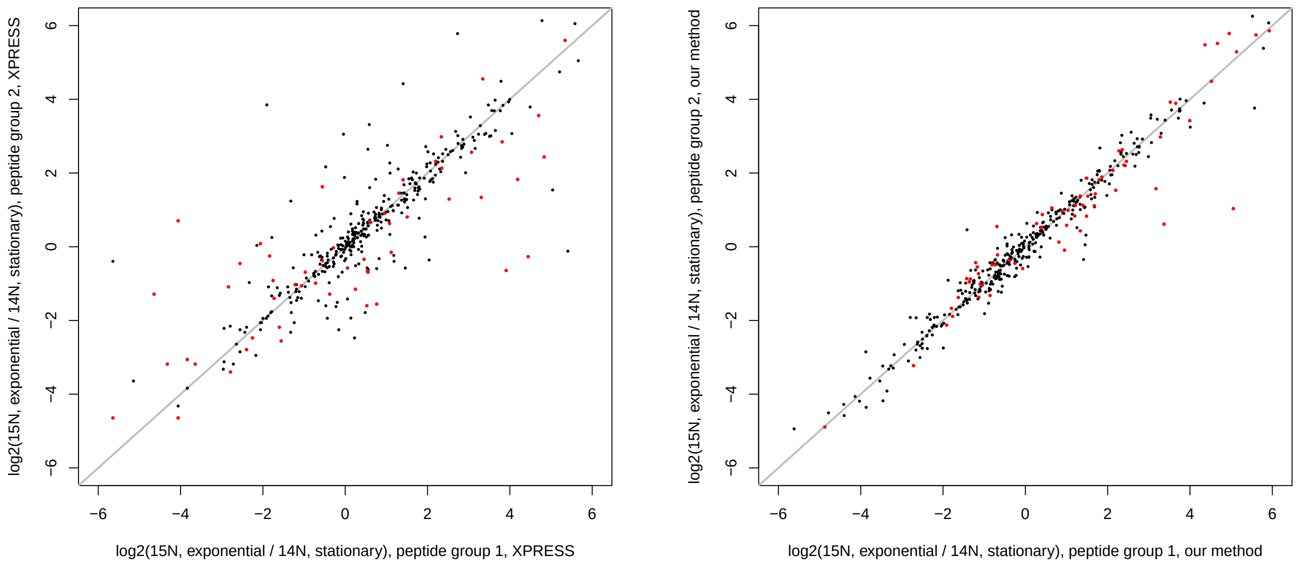

(a) (b)

Figure S2

Correlation of $\log_2$ ratios between distinct peptides from the same protein obtained by (a) XPRESS and (b) our method for the label-swap experiment. Multiple measurements (e.g. charge states) of the same peptide, when present, were collated and given a median $\log_2$ ratio prior to forming the peptide groups. Black points designate proteins identified and quantified by both methods, and red points designate proteins identified and quantified by each method specifically. Perfect correlation is designated by the diagonal gray line.

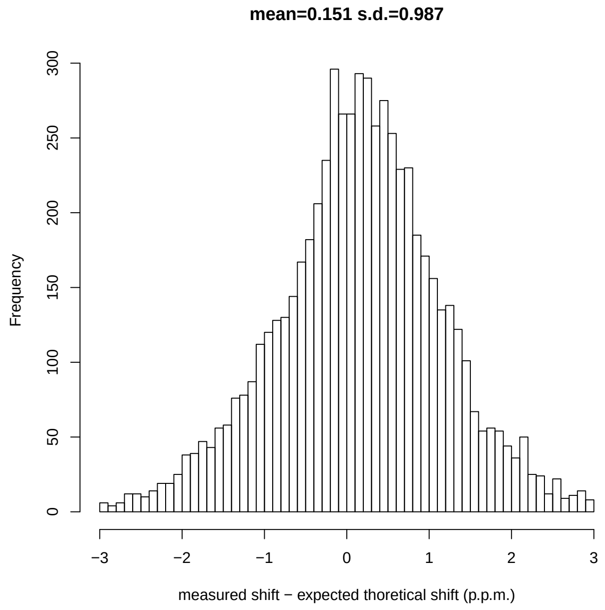

Figure S3

Precision of the data collected in the label-swap experiment. A histogram of measured shifts and expected theoretical shifts in parts per million (p. p. m.) between XIC pairs that were assigned a sequence at an FDR of 1%.

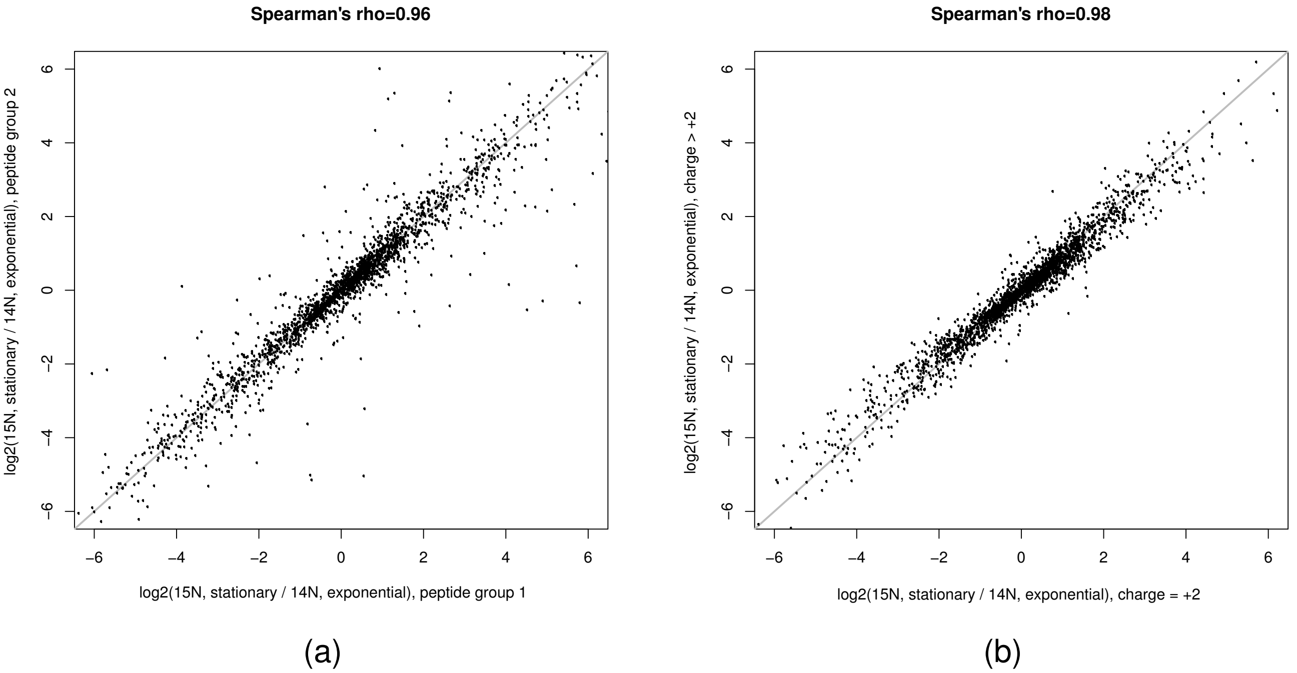

Figure S4

(a) Correlation of $\log_2$ median ratios between distinct peptides separated into two groups from the same protein samples after solubility and SCX fractionation. (b) Correlation of $\log_2$ stationary vs. exponential ratios comparing charge isoforms after solubility and SCX fractionation.

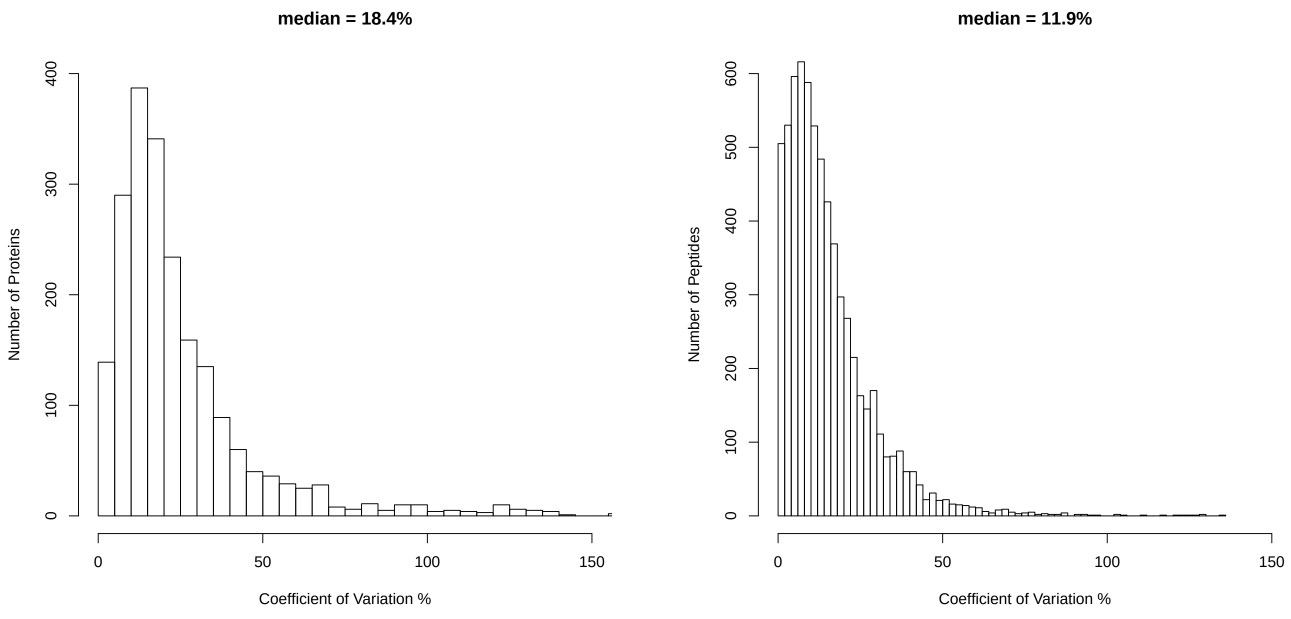

(a) (b)

Figure S5

(a) shows a histogram of the intra-experiment coefficient of variation (CV) estimated using pooled ratios of distinct peptides from each protein from the solubility and SCX fractionated data set. Multiple measurements (e.g. charge states and replicate measurements across fractions) of the same peptide, when present, were collated and given a median $\log_2$ ratio prior to computing the CV for a protein. (b) shows a histogram of the CV estimated using pooled ratios of peptides observed in multiple SCX or solubility fractions or with differing charge states.
